# Intraoperative brachial plexus injury during emergence following movement with arms restrained: a preventable complication?

**DOI:** 10.1186/1754-9493-1-8

**Published:** 2007-12-19

**Authors:** Mark H Chandler, Laura DiMatteo, Erik A Hasenboehler, Michael Temple

**Affiliations:** 1 Department of Anesthesiology, Denver Health Medical Center, 777 Bannock Street, Denver, CO, 80204-4507, USA; 2 Department of Orthopedics, niversity of Colorado Health Sciences Center, 2631 East 17th Avenue, Academic Office1, Room 4602, Aurora, CO, 80045, USA; 3 Department of Orthopedics, Spital Oberengadin, Chirurgische Abteilung, 7503 Samedan, Switzerland

## Abstract

**Background:**

Despite considerable analysis and preventive strategies, brachial plexus injuries remain fairly common in the perioperative setting. These injuries range from brief periods of numbness or discomfort in the immediate postoperative period to, in rare cases, profound, prolonged losses of sensation and function. We present a case of an orthopedic surgery patient who suffered a brachial plexus injury while under anesthesia after trying to sit upright with his arms restrained.

**Case presentation:**

After the uneventful placement of an intramedullary tibial nail, an 18 year old patient tried to sit upright with his arms restrained while still under the influence of anesthesia. In the immediate postoperative period, the patient complained of a profound loss of sensation in his left arm and an inability to flex his left elbow, suppinate his arm, or abduct and rotate his shoulder. Neurological examination and subsequent studies revealed a C5-6 brachial plexus injury. The patient underwent range of motion physical therapy and, over the next three months, regained the full function and sensation of his left arm.

**Conclusion:**

Restraining arms during general anesthesia to prevent injury remains a wise practice. However, to avoid injuring the brachial plexus while the arms are restrained, extra caution must be used to prevent unexpected patient movement and to ensure gentle emergence.

## Background

Peripheral nerve injury (PNI) remains a fairly common occurrence in the perioperative setting. An analysis of the American Society of Anesthesiology's Closed Claims Database (CCD) from 1990–1999 revealed that among 4183 closed claims, 670 (16%) were for nerve injury [1]. This percentage has remained fairly constant over the last two decades, as the previous CCD analysis in 1990 revealed a similar percentage of nerve injury claims (15%) [2]. In the more recent analysis, brachial plexus injuries (20%) ranked second only behind ulnar nerve injuries (28%), but ahead of lumbrosacral (16%) and spinal cord injuries (13%). Despite these numbers, the actual incidence of peripheral nerve injury in the perioperative setting is unknown, and likely underestimated [3]. The incidence of subclinical nerve injury may be as high as 87% in cases involving median sternotomy [4].

We present a case of an orthopedic surgery patient who suffered a brachial plexus injury while under anesthesia after trying to sit upright with his arms restrained.

## Case presentation

An 18-year-old, 63 kg otherwise healthy male was scheduled to undergo removal of hardware and placement of an intramedullary nail in his left tibia for a peri-implant tibial spiral fracture. The patient was seen in the preoperative area and the anesthetic options were discussed. After administering 2 mg of midazolam and 200 mcg of fentanyl (in divided doses) through the patient's IV in his left forearm, the patient was transported by gurney to the operating room and transferred to the operating room table. The patient's arms were placed palm up on padded arm boards, slightly abducted approximately 60 degrees from the main axis of his body. Both arms were secured to arm boards by means of two-inch "hook and loop" straps (similar to VELCRO^®^) affixed loosely over the patient's forearms. Standard anesthesia monitors were then placed (blood pressure cuff on the right arm) and after preoxygenation, the patient was induced by means of a rapid sequence intubation using lidocaine, succinylcholine, and propofol.

The patient underwent an uneventful surgical removal of hardware from his left tibia and placement of an intramedullary nail; the entire surgical procedure lasted 2 hours, 20 minutes. General anesthesia was maintained with desflurane (MAC varied from 4.8 – 7.1 throughout the case), dilaudid (3 mg in divided doses), and fentanyl (200 mcg were administered, in divided doses, throughout the remainder of the case). Seven mg vecuronuim were administered 10 minutes into the case after the patient's twitches returned from the succinylcholine, but no subsequent doses were given. Blood loss for the case was 600 cc and fluid replacement consisted of 2200 cc of lactated ringers solution. The patient maintained a blood pressure ranging from 99/35 (induction) up to 150/76 (emergence), but generally averaged about 120/65 throughout the case. Heart rate ranged from 60 bpm (induction) to 95 bpm (emergence), but averaged about 70 bpm throughout the case. The patient was warmed with an upper body Bair hugger^® ^tied loosely around the patient's wrists, and the patient's temperature ranged from 36.0 to 36.6.

Just as the surgeons were casting the patient's left leg, while the patient was still intubated (but breathing spontaneously) and receiving a MAC (Minimum Alveolar Concentration) of 3.2 desflurane (per Respiratory Gas Monitor), the patient unexpectedly tried to sit upright, coming to about a 45 degree angle relative to the horizontal plane of the operating table. This sudden movement, with both arm straps still in place, caused both arms to be rotated and extended posteriorly (like a runner breaking through a finish line tape). The anesthetist gently returned the patient to a supine position, administered 150 mcg of fentanyl in divided doses, and then gently awakened the patient about 10 minutes later once the casting was complete and postoperative films were read. The patient was extubated uneventfully after suctioning, transferred to a gurney and taken to the Postoperative Care Unit (PACU). The patient remembered nothing about the surgery, or about trying to sit upright (and the patient would continue to deny any recollection of the surgery or his attempt to sit upright even several months after the surgery).

The immediate postoperative course was uneventful; however, about 20 minutes after arrival the PACU nurse noted that the patient was not moving his left arm. The anesthesia attending was called to the bedside and a quick exam of the patient revealed an inability to lift his left arm from across his lap, abduct his shoulder, or flex or suppinate his arm. Sensation was diminished throughout his left arm, but particularly absent over his forearm, the posterior portion of his hand, and his thumb, middle and index fingers. Both neurosurgery and the microsurgical hand service were consulted and an exam of the patient's left arm revealed the following: elbow flexion 0/5; shoulder abduction and rotation 0/5; supination 0/5; all other muscle groups 5/5; diminished sensation as above. The patient was diagnosed with a C5-6 brachial plexus injury.

As an inpatient, the patient's sensation and motor function improved slightly over the next 48 hours. Imaging studies were obtained on postoperative days two and three, to include 4 views of the left shoulder, 6 views of the cervical spine, and an MRI of the cervical spine and left brachial plexus. All studies revealed normal anatomy with no signs of injury. Both physical therapy and occupational therapy were consulted on postoperative day two, and the patient was instructed to perform range of motion exercises while at home. The patient was discharged from the hospital on postoperative day 4.

On postoperative day 20 the patient reported minimal improvement in strength or sensation. Upon exam, the diminished sensation and muscle function noted at discharge appeared for the most part unchanged and atrophy was noted of the deltoid, supraspinatus and infraspinatus muscles. On postoperative day 45, the patient reported significant improvement in motor function and sensation of his left arm near symmetric to his right arm. On postoperative day 84, the patient reported that his symptoms had all but completely resolved, and he had returned to racing motocross.

## Discussion

The three principal mechanisms that result in PNI are laceration, compression, and stretching. Laceration is the least common cause, accounting for only about 30% of PNI cases [5]. Nerve compression, with consequent ischemia and actual mechanical crush injury in extreme cases, can occur in a number of perioperative settings. But nerves are surprisingly resistant to compression. Research has shown that 45–60 minutes of direct compression over a nerve is required to cause a transient conduction block, and distal nerve conduction is slowed only after three hours of compression at 250 mm Hg [6].

**Figure 1:**
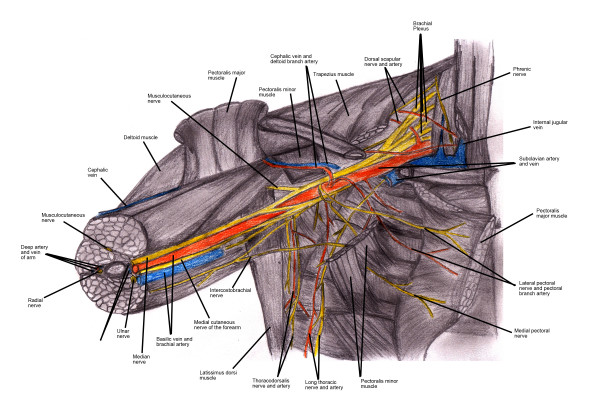
**Anatomy of the brachial plexus**. The brachial plexus is anchored proximally to the vertebral and prevertebral fascia and distally to the axillary sheath. Thus, when severe traction is placed on the neck and arm, the brachial plexus can be stretched and potentially injured.

Stretching remains the most likely culprit in most cases of perioperative PNI. While a collagenous endoneurium contributes to the elasticity of nerves, this elasticity can be exceeded, with concomitant injury and, in extreme cases, avulsion [5]. The anatomical relationship to surrounding anatomic structures makes the brachial plexus particularly vulnerable to stretch injuries. Proximally the brachial plexus is anchored to vertebrae and prevetebral fascia, while distally it is similarly anchored to the axillary sheath (see Figure 1). Thus, while other neuronal bundles may slide within their surrounding tissues, the brachial plexus must stretch, sometimes to the point of injury, as traction is placed on the shoulder and neck [7].

Our understanding of how peripheral nerves repair themselves has increased significantly in the last few decades with advances in cellular and molecular biology. Unlike the central nervous system, where injury is circumvented by strengthening and reprogramming uninjured pathways, repair in the periphery often occurs over preexisting pathways, involving cellular bodies in the spinal cord and ganglia, as well as Schwann cells, macrophages and inflammatory cell [5].

Various methods of classifying nerve injuries have emerged over the years, but among the most enduring is the neurapraxia/axonotmesis/neurotmesis model developed by Seddon, later modified to five tiers by Sunderland (see Table 1). In neurapraxia (aka 1^st ^degree nerve injury by Sunderland), there is no loss of neural continuity and only minor alterations in the myelin sheath. This produces a transient loss of function, likely due to a local ion-induced conduction block. In axonotmesis (2^nd ^degree), there is interruption of the axon and myelin, but all other tissues such as the endoneurium, epineurium, and perineurium are intact. This surviving tissue framework aids recovery by guiding neuron and axonal growth. There is still some chance of functional recovery in 3^rd ^degree injury (a class Sunderland places between axonotmesis and neurotmesis), which includes damage to the endoneurium in addition to the axon and myelin sheath, with preservation of the epineurium, and perineurium. Neurotmesis, or dissection of the nerve, is further divided by Sunderland into two classes depending on whether the epineurium is preserved (4^th ^degree) or severed (5^th ^degree). In the case of neurotmesis, rarely is any degree of recovery possible without surgery [5].

**Table 1:** Seddon's and Sunderland's classifications of nerve injuries

**Injury**	**Pathophysiology**	**Exam Findings**	**Nerve Studies**	**Prognosis**
Neurapraxia (Seddon) *First Degree (Sunderland)*	Reversible conduction block. Local compression with ischemia; selective demyelination of the axon sheath possible.	Motor paralysis: complete Muscle atrophy: minimal Sensory alteration: minimal, often with sparing	Distal nerve conduction: present. Motor unit action potential: absent. Fibrillation: occasionally detectable.	Good prognosis. Full recovery usually within days to 2–3 weeks
Axonotmesis (Seddon) *Second Degree (Sunderland)*	More severe injury with disruption of the axon and myelin sheath.	Motor paralysis: complete Muscle atrophy: progressive Sensory alteration: complete	Distal nerve conduction: absent. Motor unit action potential: absent. Fibrillation: present.	Fair prognosis. Full recovery possible without surgery; recovery at 1 mm/day
*Third Degree (Sunderland)*	Endoneurium disrupted; epineurium and perineurium intact.	Same	Same	Same
*Fourth Degree (Sunderland)*	Endoneurium and perineurium disrupted; epineurium intact.	Same	Same	Same
Neurotmesis (Seddon) *Fifth Degree (Sunderland)*	Complete nerve division with disruption of the endoneurium, perineurium, and epineurium.	Same	Same	Poor prognosis. Requires surgery with varying degrees of impairment present even after surgery

A number of clinical findings have been sited as possible predisposing conditions to peripheral nerve injuries, to include hypothermia, diabetes, coagulopathy, and induced hypotension among many others [7].

Over the years, various mechanisms in the perioperative setting have been implicated in brachial plexus injuries, to include automated blood pressure cuffs in very thin patients [8], patient positioning (especially in obese patients) [7], and even a falling arm board with a patient's arm strapped down [9]. Open-heart surgery is still the most common setting for perioperative brachial plexus injury, inviting a considerable amount of research and literature in an effort to prevent this problem. Particular attention has been paid to median sternotomy with retraction and harvesting the Internal Mammary Artery (IMA), where both traction and a fractured first rib may play a role in injuring the brachial plexus [4]. No case, to our knowledge, has been reported of a brachial plexus injury from an unexpected attempt to sit upright with arms strapped in place. It seems clear from review of other cases of brachial plexus injury, and an appreciation of the anatomy and forces at work, that such movement in a restrained patient could put considerable traction on the brachial plexus.

Fortunately, the prognosis for intraoperative brachial plexus injury is fairly good. In a review of 22 cases of intraoperative brachial plexus injury, all patients eventually recovered to a significant extent even when recovery was not complete, and none suffered late deterioration or chronic pain. Interestingly, the median full recovery took 10 weeks in open-heart surgery patients and 20 weeks in weeks in noncardiac surgery patients [10].

## Conclusion

In summary, we presented a case of brachial plexus injury from unexpected movement in a patient with arms restrained. While hook and loop straps may have contributed to this patient's injury (as they likely did in the case of the falling arm-board), these restraints can also prevent the arms from sliding off arm boards and causing injury. Thus, while restraining a surgical patient's arms remains a wise practice in most cases, caution should be exercised in preventing patient movement and carefully controlling emergence.

## Abbreviations

BPI: Brachial Plexus Injury

PNI: Peripheral Nerve Injury

PACU: Post Anesthesia Care Unit

MAC: Minimum Alveolar Concentration

CCD: Closed Claims Database

MRI: Magnetic Resonance Image

## Competing interests

The author(s) declare that they have no competing interests.

## Authors' contributions

MHC wrote, researched, edited, and submitted the article, and served as the anesthesia attending during the actual case.

LD was the orthopedic resident who performed the tibial surgery during the actual case. She also performed much of the research on nerve injuries and created the table outlining the Seddon and Sunderland nerve injury classifications.

EAH drew the illustration of the anatomy of the brachial plexus.

MT performed the anesthetic during the actual case, and his anesthetic record, along with personal recollections, formed the basis for the "presentation" portion.

All authors read and approved the final manuscript.
